# Magnetic resonance imaging in the management of Crohn’s disease: a systematic review and meta-analysis

**DOI:** 10.1186/s13244-021-01064-9

**Published:** 2021-08-18

**Authors:** Rani Ahmad, Amr M. Ajlan, Ayman A. Eskander, Turki A. Alhazmi, Khalid Khashoggi, Mohammad A. Wazzan, Ahmed H. Abduljabbar

**Affiliations:** 1 Department of Radiology, Faculty of Medicine, King Abdulaziz University, Jeddah, Saudi Arabia; 2 Department of Medicine, Umm Al Qura University, Makkah, Saudi Arabia

**Keywords:** Magnetic resonance imaging, Radiation, Crohn disease, Treatment

## Abstract

**Objectives:**

Crohn’s disease (CD) is a condition that can occur in any part of the gastrointestinal tract, although usually forms in the colon and terminal ileum. Magnetic resonance imaging (MRI) has become a beneficial modality in the evaluation of small bowel activity. This study reports on a systematic review and meta-analysis of magnetic resonance enterography for the prediction of CD activity and evaluation of outcomes and possible complications.

**Methods:**

Following the PRISMA guidelines, a total of 25 low-risk studies on established CD were selected, based on a QUADAS-II score of ≥ 9.

**Results:**

A sensitivity of 90% was revealed in a pooled analysis of the 19 studies, with heterogeneity of *χ*^2^ = 81.83 and *I*^2^ of 80.3%. Also, a specificity of 89% was calculated, with heterogeneity of *χ*^2^ = 65.12 and *I*^2^ of 70.0%.

**Conclusion:**

It was concluded that MRI provides an effective alternative to CT enterography in the detection of small bowel activity in CD patients under supervision of radiologist for assessment of disease activity and its complications. Its advantages include the avoidance of radiation exposure and good diagnostic accuracy.

## Key points

- MRI may be considered as an effective diagnostic technique in moderate to severe CD patients.
- It plays an increasingly important role as non-invasive and effective method to evaluate the small-bowel involvement and the possible intestinal and extra-intestinal complications, in patients affected by CD.
- MARIA score found it to be the most sensitive and specific tool.

## Introduction

Crohn’s disease (CD) is a form of inflammatory bowel disease (IBD), with an approximate frequency of 20.2 per 100,000 person-years in North America, 12.7 per 100,000 person-years in Europe, and 5 person-years in Asia [1, 2]. Broadly, one-third of CD patients have either colitis, ileocolitis, ileitis, penetrating, or stricturing intestinal (in 50% of cases) complications at the time of diagnosis [3]. While the pathology and etiology of this type of IBD are elusive, a dysregulation of the mucosal immune response is thought to play an important role in the pathogenesis of the disease, together with environmental and genetic factors [4].

An analysis of the medical history and a physical examination of the patient is the first step towards the diagnosis of CD, supported by pathologic, endoscopic, and laboratory data, using a variety of clinical severity indices, such as the Crohn's Disease Activity Index (CDAI), Harvey and Bradshaw Index, Simple Index, Oxford Index, van Hees Index, and Cape Town Index. Clinical disease activity is categorized by the European Crohn’s and Colitis Organization (ECCO) into severe, moderate, and mild [5]. While disease activity generally follows a tripartite classification, there is neither a standardized system of grouping nor a canonical definition of levels. The CDAI is perhaps the most useful and widespread research tool for quantifying the level of disease activity. A score of > 220 is used to recruit patients with active Crohn’s disease in many clinical trials, indicating a moderate to severe disease activity [5].

The Montreal and Paris classifications of CD group patients based on localization, behavior, growth, and onset; the behavior is subdivided into penetrating, non-stricturing/non-penetrating, and stricturing [6]. The most severe prognoses are usually found in the perianal and penetrating form of the disease in comparison with other types [7]. The clinical importance of differentiation according to subtype is shown during the active inflammatory phase of CD, where treatment requires modification when there is a coexistence of extramural disease or if associated fibrostenotic disease occurs with obstructive symptoms [8]. Accordingly, CD is classified clinically into four subtypes: reparative or regenerative (tending to be characterized by regeneration), inflammatory, fibrostenotic, and fistulizing or perforating [9].

There is no consensus about the preferred MRE score and they are not routinely utilized. Several MRE-based indices are common for analysis, including the Clermont score, the magnetic resonance index of activity (MaRIA), the Crohn’s disease magnetic resonance imaging index (CDMI), the Lemann index, and the magnetic resonance enterography global score (MEGS) [10–13]. This systematic review and meta-analysis focus on the use of MRE to evaluate the types of disease behavior, and to use this information to find possible correlates with clinical findings. This review correlated different MRE methods (Clermont score, MaRIA, CDMI, MEGS) for Crohn disease activity assessment.

## Materials and methods

### Design and eligibility criteria

This mixed-studies review incorporates a systematic search strategy and quality appraisal method, and is based on the preferred reporting items for systematic reviews and meta-analysis (PRISMA) guidelines for researchers. A comprehensive search strategy was carried out using electronic databases, such as EMBASE, CINAHL, Cochrane Central Register of Controlled Trials, and MEDLINE. All forms of study designs were included with the exception of qualitative studies and management reviews, since the main focus of the review was the quantitative assessment of MRI. Also, all relevant studies were included that examine disease type and activity in patients with CD where MRI was involved as a diagnostic tool. Patients with an established diagnosis of CD were the target population.

Prospective and retrospective studies that were given focus examined subtypes of CD, detection and characterization of the disease using MRE, diagnostic modality (surgery, histopathology, endoscopy, ileocolonoscopy, etc.), and patient population (i.e., established diagnosis of CD). No restrictions were made regarding country, patient’s age, or gender. The review is restricted in terms of the written language of articles, only including those in English. The search was limited to articles published from 1 January 2000 to 1 October 2019.

### Extraction

Two independent reviewers reviewed the retrieved studies. Any disagreements were resolved either by a third reviewer or through a consensus approach. The two reviewers extracted the data independently from the selected studies. The data extraction method was established prior to the study commencement and included information on imaging characteristics, such as bowel preparation, radiologist experience, oral and intravenous contrast, MRI strength, and time interval. Also, information on study characteristics were included, comprising age, gender, number of patients, type of study, reference standard, patient population, location studied, year, and country. Particular values were calculated or extracted for the selected studies which provided data regarding individual patients, such as false positive, false negative, true positive and true negative. All outcomes used in the selected articles regarding MRI, CD, clinical examinations, and imaging characteristics were considered as primary for this review. The following keywords were used: (disease type OR behavior type) AND (clinical subtype OR DWI classification OR Clermont Score OR category) AND (Montreal OR Paris) OR (MARIA OR MRI activity index) AND Crohn’s disease.

### Risk of bias individuals

The Quality Assessment of Diagnostic Accuracy Studies (QUADAS-II) tool developed for diagnostic studies was used to assess the risk of bias, applicability, and quality. Studies with scores greater than 9 were classified as low risk.

### Data analysis

A summary of the results exhibits the effectiveness of MRE in detecting activity, including MARIA, using sensitivity and specificity values for studies that did not provide raw data. For studies that provided raw data, contingency tables were constructed using true positive, true negative, false positive, and false negative information. Likelihood ratios, sensitivity, specificity, and 95% confidence intervals were calculated for each study. Summary receiver-operating characteristic (ROC) curves and forest plots were presented through graphical representation, which were made using the Comprehensive Meta-Analysis software (CMA), version 3 by Biostat, Inc. For a pooled analysis, 0.5 was added to all cells that comprised a value of 0 to include all studies in the analysis. The *I*_2_ test was used to assess the heterogeneity.

## Results

The preliminary database search yielded 2567 articles. These were reduced further by sorting according to title, resulting in a yield of 252 articles; these were then further reduced to 35 articles based on an appraisal of abstracts. A total of 25 articles fulfilled the inclusion criteria after consideration of the full articles and following contact with authors for missing information [1–4, 12, 14–30]. Of the 25 included articles, 7 were retrospective studies, 10 were prospective studies, and 8 were unclear with respect to type. The majority of the prospective studies used consecutive patients to restrict the selection bias. These gave sufficient information to constitute a pooled analysis; for instance, 10 studies of the initial 35 selected articles offered only summaries of results. All studies were assessed as low risk, based on QUADAS-II scores of ≥ 9.

In the 25 articles that met the inclusion criteria, a total of 2120 patients took part, out of which, 1470 patients were incorporated into a meta-analysis. The largest sample size among those patients included in the pooled analysis was 305. In an examination of the data of these 1470 patients, 150 underwent endoscopy and MR within 30 days to evaluate the distal ileum condition, which resulted in an MRI pooled specificity of 80% and a sensitivity of 88% in predicting CD activity.

### Correlation between MRE and Crohn disease type and subtype

A sensitivity of 61% for predicting disease type or subtype was revealed in a pooled analysis of 19 studies [1, 2, 4, 12–14, 16–24, 31–33] with a heterogeneity of *χ*^2^ = 61.38 and *I*^2^ of 60.7%. A specificity of 59% was reported for the pooled analysis with a heterogeneity of *χ*^2^ = 65.12 and *I*^2^ of 70.0%. The negative predictive ratio was 0.21 and the positive predictive ratio was 6.1 using a random effects model.

Aphthous ulcers in the penetrating type of CD is demonstrated in MRE studies as a high signal intensity nidus, covered by moderate signal intensity area best seen on high-resolution SSFP images with fat suppression [34]. Oral contrast material can outline the transmural ulcers, and thus be observed as linear high-signal intensity seen passing through the gastrointestinal tract. For more accurate evaluation of transmural lesions, along with the associated peri-intestinal inflammatory changes, images must be made on a plane that is perpendicular to the bowel [9]. The penetrating disease that has large sinus tracts with or without fistulas can be evaluated using oral contrast material. This outlines these tracks, enabling them to be seen as high-signal intensity lines that pass through the full thickness of the wall of the bowel to form a small collection adjacent to it, or communicated through an adjacent luminal structure, such as the urinary bladder or another bowel segment [35].

Magnetic resonance enterography (MRE) can detect fibrostenotic disease of stricturing type, observed as a fixed narrowing of the affected segment [36, 37]. Both T1- and T2-weighed images can demonstrate chronic fibrotic strictures, showing inhomogeneous contrast enhancement in post-IV contrast sequences but without the surrounding inflammation of mesentery or obvious edema [36, 38]. The absence of gut motility in active or chronic CD is observed using fast cine sequence (T2-weighted SSFP or echo planar imaging techniques). These images should be obtained before administering the spasmolytic drug, which improves evaluation of the motility of the bowel and enables differentiation between fibrotic and inflammatory strictures [9].

The diagnostic power of the markers was determined through a logistic regression analysis when differentiating between the Montreal behavioral classes (Table 1). Small bowel CD type and various subtypes were diagnosed and presented through forest plots based on sensitivity, specificity, and ROC curves with respect to the use of MRE. The aggregate sensitivity and specificity of MRE in assessing CD type and subtype was 67.78% and 61.88%, respectively, while the aggregate sensitivity and specificity for endoscopy was 64.15% and 62.05%, respectively.

**Table 1:** Sensitivity and specificity of predicting disease type or subtype

CD type/subtype	MRE sensitivity (%)	MRE specificity (%)	Logistic regression	Important differentiating MRI feature
Penetrating type	63.7	60.3	*p* < 0.001	Presence of ulcer on T2-weighted images (T2WIs) [9]
Nonstricturing/non penetrating	58.2	48.4	*p* < 0.001	Presence of the classical CD features without complications on T2-weighted and gadolinium-enhanced T1-weighted sequences [8]
Stricturing type	63.9	63.2	*p* < 0.001	Presence of segment(s) of luminal narrowing (fibrotic or inflammatory cause) on unenhanced T2-weighted and gadolinium-enhanced T1-weighted sequences [8]
Inflammatory subtype (Local inflammation, aphthoid and deep ulcers, frequent transmural inflammation with lymphoid aggregates, and granuloma development define the active inflammatory subtype of CD)	73.8	64.9	*p* < 0.001	Acute inflammation is best seen in T2WIs in fat suppressed sequences; stratified contrast confirms CD is active [9]
Fibro stenotic subtype (persistent intestinal damage, chronic inflammation of the gut wall tends to proceed to fibrostenotic and irreversible consequences (bowel strictures and blockage))	81.5	63.3	*p* < 0.001	Presence of stenotic segment that persist in the cine images and shows hypointense on T1WIs and T2WIs, and on contrast enhancement shows inhomogenous delayed enhancement [9]
Reparative or regenerative subtype	72.4	64.2	*p* = 0.005	Luminal narrowing may be seen without any sign of obstruction. Post contrast T1 images showed homogenous continuous enhancement [39]
Fistulizing/perforating subtype (The presence of deep penetrating ulcers, which can lead to the formation of sinus tracts, fistulas, and/or abscesses, characterises it)	61.0	68.9	*p* = 0.008	Presence of fistula or signs of perforation with adjacent collection, best seen on gadolinium-enhanced T1-weighted sequences [9]

### Correlation of MRI with disease activity

The studies presented in Table 2 were added to perform the indices revalidation. Variable outcomes have been shown by validating the studies on the ability of the indices to detect active disease. By contrast, there is considerable inconsistency between gold standards, evaluated bowel segment, and validating studies’ methods, making it difficult to draw firm conclusions on the capacity of these indices to reflect disease activity levels.

**Table 2:** Sensitivity and specificity of detecting CD activity

MRE method of assessing Crohn disease activity	MRE sensitivity (%)	MRE specificity (%)	Number of patients	Studies	Meta-analysis results
Clermont score	79	73	50	[24, 27]	*p* < 0.001
MaRIA	85	79	42	[40]	*p* < 0.003
CDMI	78	74	16	[26]	*p* < 0.001
MEGS	75	71	21	[28]	*p* < 0.007

*MaRIA* Magnetic resonance index of activity, *CDMI* Crohn Disease MRI Index, *MEGS* Magnetoencephalography

Small bowel CD activity determined through the use of MRE was presented through forest plots based on sensitivity, specificity, and ROC curves. The aggregate sensitivity and specificity of MRE in assessing CD activity was 79.25% and 74.25%, respectively.

## Discussion

Thia et al. based study on the evolution of Crohn's disease reported 19% of patients with known penetrating or stricturing complications within the first 90 days of diagnosis based on Montreal classification, while patients diagnosed with non-stricturing and non-penetrating disease at baseline had intestinal complication on progression, 76.7% of these patients required bowel resection surgery [3].

Penetrating disease, hospitalization for flares or consequences of the illness, surgery, extra-intestinal manifestations (EIMs) affecting two systems, or poor response to presently available medications are all signs of aggressive CD [41].

The current systematic study is the largest systematic study of MRI for small bowel identification in CD. Previous research and reviews looked at the sensitivity and specificity of MR imaging in detecting small bowel movement [42, 43]. We focused only on the small bowel.

The gold standard diagnostic approach for assessing intra- and extra-luminal CD complications is MR imaging (Fistula, abcess and stenosis). For the identification of stenosis, we discovered a high specificity with a moderate sensitivity. Previously, studies found a high detection rate for stenosis in both small and large bowel illness [24, 44] CT imaging may be beneficial, according to Qiu et al. for fistulas and stenosis detection but with no statistical significance and sensitivity and specificity were comparable to published studies [45, 46]. Qiu et al. found results for stenosis that were similar to ours (sensitivity 65.3%, specificity 94.4%).

The lack of uniformity of imaging indications suggestive of ongoing illness, especially with the expanding number of sequences available, is one of the challenges in replacing the gold standard with MR imaging. Previous research has shown that wall enhancement, mucosal lesions, and wall T2 hyperintensity are the most reliable indications of inflammation for MR [43, 44]. Additionally, a study by Udayasankar et al. found similar results in both the small and large bowel [45]. Diffusion-weighted MRI sequences have also been demonstrated to have good sensitivity and specificity in previous investigations [46, 47]. Recently, validated scoring systems have been developed, such as the MaRIA (Magnetic Resonance Index of Activity) score for assessing disease activity and severity, and the Lemann score, or Crohn's Disease Digestive Damage Score, which considers a variety of factors (clinical, endoscopic, and imaging findings) and attempts to measure cumulative damage [48, 49].

If the results of MR imaging influence physician management, this is a significant factor. Mendoza et al. found that MR aided decision-making in more than half of patients, particularly when biological treatments and surgery were involved [50]. Messaris et al. showed that 69% of patients had changes to medical and/or surgical management after clinicians were given MR imaging results [51]. Similar results have shown that MR findings influence surgical approaches to managing Crohn's patients [52].

Many researches looked at the use of ultrasound and computed tomography as alternate imaging modalities. Ultrasound has the advantage of avoiding ionising radiation and being very affordable [53]. Previous studies assessing ultrasound have demonstrated high sensitivities and specificities. There is one large-scale trial comparing US and MR currently in progress: the UK-based MR Enterography or Ultrasound in Crohn's disease (METRIC) trial [53–55]. Similarly, one meta-analysis has shown similar accuracy between CT and MR. CT has the benefit of being widely available and cost-effective. It, however, also carries the risk of ionizing radiation, especially amongst patients who might require multiple scans throughout the course of their life-long disease [56].

## Limitations

This review may be limited in terms of the varied lengths of time between MRI and the reference standard. Thus, some of the findings might be nominally inaccurate in determining disease activity because clinical activity can change relatively quickly, particularly with the use of medication. This article could not differentiate between severity of small bowel activity and other clinical features, owing to a lack of data. A number of other studies have recommended that MRI has better association with CD severity indices [32, 40]. Also, per-segment analysis was not performed, which might have led to an overestimation of MRI accuracy. This may be a product of the use of endoscopy as a reference standard and its unsuitability in assessing added proximal small intestine. Another limitation was that only one complication was analyzed in the studies, and others were not well visualized. This review was not able to determine whether additional advanced MRI may have had an advantage over other methods owing to the small number of studies that provided relevant information.

## Conclusion

This review has shown the effectiveness of MRE in detecting CD, and illustrated its considerable accuracy in detecting disease activity: a MARIA score found it to be the most sensitive and specific tool. MRE was able to predict disease types and subtypes to a high degree of accuracy, permitting radiologists to initiate a selection of the disease type or subtype from MRI features. However, radiologists may need to provide the essential characteristics of the disease in arriving at a final diagnosis of disease types and subtypes, because the sensitivity and specificity of MRE are not high enough to determine these accurately, specifically in comparison with endoscopic techniques.

## Data Availability

The data will be available for review from the corresponding author on request.

## Abbreviations

CD: Chron’s disease
CDAI: Crohn's Disease Activity Index
CDMI: Crohn Disease MRI Index
CMA: Comprehensive meta-analysis software
IBD: Inflammatory Bowel disease
MaRIA: Magnetic resonance index of activity
MEGS: Magnetic resonance enterography global score
MRE: Magnetic resonance enterography
MRI: Magnetic resonance imaging
PRISMA: Preferred reporting items for systematic reviews and meta-analysis
QUADAS: Quality assessment of diagnostic accuracy studies
ROC: Receiver-operating characteristic
